# Hypogammaglobulinemia and Risk of Exacerbation and Mortality in Patients with COPD

**DOI:** 10.2147/COPD.S236656

**Published:** 2020-04-16

**Authors:** Are M Holm, Siw L Andreassen, Vivi Lycke Christensen, Johny Kongerud, Øystein Almås, Henrik Auråen, Anne H Henriksen, Ingeborg S Aaberge, Olav Klingenberg, Tone Rustøen

**Affiliations:** 1 Institute of Clinical Medicine, Faculty of Medicine, University of Oslo, Oslo, Norway; 2 Department of Respiratory Medicine, Oslo University Hospital, Rikshospitalet, Oslo, Norway; 3 Department of Medicine, Drammen Hospital, Drammen, Norway; 4 Division of Emergencies and Critical Care, Department of Research and Development, Oslo University Hospital, Ullevål, Oslo, Norway; 5 Lovisenberg Diaconal University College, Oslo, Norway; 6 Department of Nursing Science, Institute of Health and Society, University of Oslo, Oslo, Norway; 7 Department of Medicine, Østfold Hospital, Kalnes, Norway; 8 Department of Circulation and Medical Imaging, St. Olav’s University Hospital, Trondheim, Norway; 9 Infection Control and Environmental Health, Norwegian Institute of Public Health, Oslo, Norway; 10 Department of Medical Biochemistry, Oslo University Hospital, Oslo, Norway

**Keywords:** COPD, immunodeficiency, IGG deficiency

## Abstract

**Introduction:**

Chronic obstructive pulmonary disease (COPD) may, in some patients, be characterized by recurring acute exacerbations. Often these exacerbations are associated with airway infections. As immunoglobulins (Ig) are important parts of the immune defence against airway infections, the aim of this study was to relate the levels of circulating immunoglobulins to clinical features in unselected patients with COPD included in a Norwegian multicenter study.

**Methods:**

Clinical and biological data, including circulating levels of immunoglobulins, were assessed in 262 prospectively included patients with COPD GOLD stage II–IV at five hospitals in south-eastern Norway. A revisit was done after one year, and survival was assessed after five years. Clinical features and survival of those with immunoglobulin levels below reference values were compared to those with normal levels.

**Results:**

In total, 11.5% of all COPD patients and 18.5% of those with GOLD stage IV had IgG concentrations below reference values. These patients were more likely to use inhaled or oral steroids, had lower BMI, and lower FEV1%. Moreover, they had significantly more COPD-related hospital admissions (2.8 vs 0.6), number of prednisolone courses (3.9 vs 1.2), and antibiotic treatments (3.7 vs 1.5) in the preceding year. Importantly, hypogammaglobulinemia was significantly associated with reduced survival in a log-rank analysis. In multivariate regression analysis, we found that the higher risk for acute exacerbations in these patients was independent of other risk factors and was associated with impaired survival.

**Conclusion:**

In conclusion, our study suggests that hypogammaglobulinemia may be involved in poor outcome in COPD and may thus be a feasible therapeutic target for interventional studies in COPD.

## Introduction

Chronic obstructive pulmonary disease (COPD) is considered the third leading cause of death worldwide.^1^ Traditionally, the disease is staged by spirometry results defined by the Global Initiative for Chronic Obstructive Lung Disease (GOLD) classification of severity of airflow limitation.^2^ In the 2011 revision, a model for staging based on intensity of symptoms and the frequency of exacerbations was introduced,^3^ and it was shown that the new classification was better at predicting exacerbations in COPD patients.^4^ Aiming to better predict prognosis and to direct targeted therapy, a new classification has recently been issued, further integrating the role of airflow obstruction and disease manifestations in COPD in order to better predict prognosis and treatment response.^5^

With the same aim, such predictions have also been made using clinical criteria to define groups of patients, so called phenotypes.^6^ The classically defined phenotypes of COPD are chronic bronchitis and emphysema.^7^ In a review article from 2012 three different phenotypes were suggested: 1) overlap or mixed COPD-asthma, 2) exacerbator (two or more exacerbations annually), and 3) emphysema-hyperinflation.^8^ Specifically, frequent exacerbations are an important factor in disease development, affecting lung function decline, and also quality of life.^9^ It has been shown that although those with frequent exacerbations may be relatively few, they account for more than half of the exacerbation-related hospitalizations, which are associated with a three-fold increase in mortality.^10^ Identifying the exacerbator-phenotype, therefore, may be of clinical importance.

An exacerbation of COPD may have several causes, and COPD exacerbations have been classified into four groups termed: bacteria-predominant, virus-predominant, eosinophil-predominant, and pauci-inflammatory. Interestingly, patients tend to repeat the same kind of exacerbation,^11^ suggesting certain individual qualities in patients that lead to exacerbation, such as eosinophilia, microbial colonization, or immunodeficiency. Regarding the latter, immunoglobulin (Ig) G is the most predominant immunoglobulin in plasma, and represents about 75% of total Ig.^12^ Immunoglobulin deficiency, known as hypogammaglobulinemia, is characterized by recurrent airway infections, particularly by encapsulated bacteria. It is treated using intravenous or subcutaneous immunoglobulin replacement therapy.

Despite the similarities between airway infections in hypogammaglobulinemia and the infections in some individuals with COPD, only a few studies have explored the correlation between the manifestations of COPD and Ig levels. In a reassessment of patients included in two previous trials, Leitao Filho et al found that 18–20% of the patients had one or more IgG subclass deficiencies, and that reduced levels of IgG1 and IgG2 were associated with increased risk of acute exacerbations and hospitalizations.^13^ Finally, two smaller observational studies found that COPD patients who were on Ig-replacement treatment had fewer acute exacerbations, further suggesting a link between hypogammaglobulinemia and acute exacerbations of COPD.^14^,^15^ The aim of this study was to determine the prevalence of hypogammaglobulinemia in a cohort of stable COPD patients and to relate Ig levels to manifestations of COPD, such as lung function, frequency of exacerbations and self-reported symptoms, and to survival, with the ultimate purpose of facilitating future interventional studies using gammaglobulin replacement therapy in COPD.

## Methods

This study was part of a larger study, termed “Symptom Clusters and Immune Markers in Patients with COPD”.^16^ Patients with stable COPD were consecutively included at three outpatient clinics and one referral hospital in the South Eastern region of Norway, and clinical and biological data were registered. Patients were included if they were >18 years of age, were diagnosed with stage II–IV disease using the GOLD criteria,^2^ were able to read and understand Norwegian, and had no cognitive impairment. Patients who had pulmonary infection, acute exacerbation, or cancer at the time of evaluation were excluded. Written informed consent was obtained from all patients.

At enrollment, patients were asked to complete study questionnaires regarding symptoms, demographics, and comorbidities.^17^ The St. George’s Respiratory Questionnaire (SGRQ) was used to measure quality of life. A change in the total score of four was regarded a clinically meaningful.^18^ Body mass index, number of years smoking, and number of years since diagnosis of COPD were registered and medical records were reviewed for disease and treatment information. The modified Medical Research Council (mMRC) Dyspnea Scale was used to assess dyspnea severity.^19^ At enrollment, all patients underwent pulmonary function tests (PFTs), such as forced expiratory volume in one second (FEV1) and forced vital capacity (FVC), with predicted values calculated according to the guidelines of the European Respiratory Society.^20^ Number of hospital admissions, prednisolone courses, and antibiotic treatments in the previous year were registered, but precise time of these signs of exacerbation was not registered. Disease severity was classified using the GOLD criteria.^2^,^3^ For this calculation, the number of prednisolone courses (self-reported) in the last 12 months was used as a measure of the number of exacerbations. Six minute walk test (6MWT) was performed according to standard procedures. Blood gas analyses, plasma immunoglobulin analyses, and a chest X-ray were performed at inclusion. In the first 180 consecutively included patients, pneumococcal antibody titers and IgG subclass concentrations were measured. IgG, IgA, and IgM were quantified by turbidimetry on a Roche Modular P instrument (Roche, Switzerland) with reagents from Roche (Oslo University Hospital) or on a similar instrument from Abott (Østfold Hospital) or a Dimension Vista instrument from Siemens Healthcare (Bærum Hospital). IgG subclasses were measured by immunonephelometry on a ProSpec instrument (Siemens Healthcare Diagnostics, Munich, Germany) with reagent kits from Siemens. Patients with IgG levels below reference values (6.1–14.9 g/L) were termed hypogamma-COPD. Pneumococcal antibody levels given as arbitrary units (U/mL) to a mix of 23 serotypes were measured using enzyme linked immunosorbent assay (ELISA) after CWPS adsorption of sera.^21^

Patients who participated in the study were summoned to a one-year follow-up, and 185 patients attended. All tests and questionnaires were repeated at follow-up, including blood tests and X-rays. For survival assessment, data from the civic registration system in Norway were obtained five years after inclusion of the last patient (September 1, 2017), but cause of death was not available.

The Regional Committees for Medical and Health Research Ethics, the Norwegian Directorate of Health and the privacy ombudsman at Oslo University Hospital approved this study (approval no. S-09102a 2009). The study was registered at ClinicalTrials.gov with the identifier NCT01016587. The study was conducted according to the Declaration of Helsinki.

Independent Student’s *t*-tests, Mann–Whitney *U*-tests, and Chi-squared tests were used to evaluate differences between the groups. To explore independent predictors of two or more COPD exacerbations per year, a logistic regression model was fitted using backwards elimination retaining variables with a significance level above 10%. The following factors were included in the multivariate regression analysis: age, gender, COPD grade, current smoking, BMI, use of inhaled steroids, FEV1% predicted, years since diagnosis, hyperinflation on X-ray, daily cough, bowel and rheumatic disease. P-values of <0.05 were considered statistically significant. PASW Statistics 22 (SPSS Inc., Chicago, IL, USA) was used to perform statistical analysis. For the multivariate regression analysis, STATA (StataCorp LP, College Station, TX, USA) was used. Latent class analysis (LCA) has previously been used to identify subgroups of patients included in this study based on physical and psychological symptoms.^16^ For comparisons of transplant-free survival between groups log rank test was applied using STATA. Anonymized raw data may be accessed by contacting the corresponding author.

## Results

In total, 267 patients were included, and plasma levels of IgG were available in 262. The five patients without known IgG values were excluded from further analyses. Severity of COPD disease according to the GOLD criteria was available in all cases.

**Figure 1:**
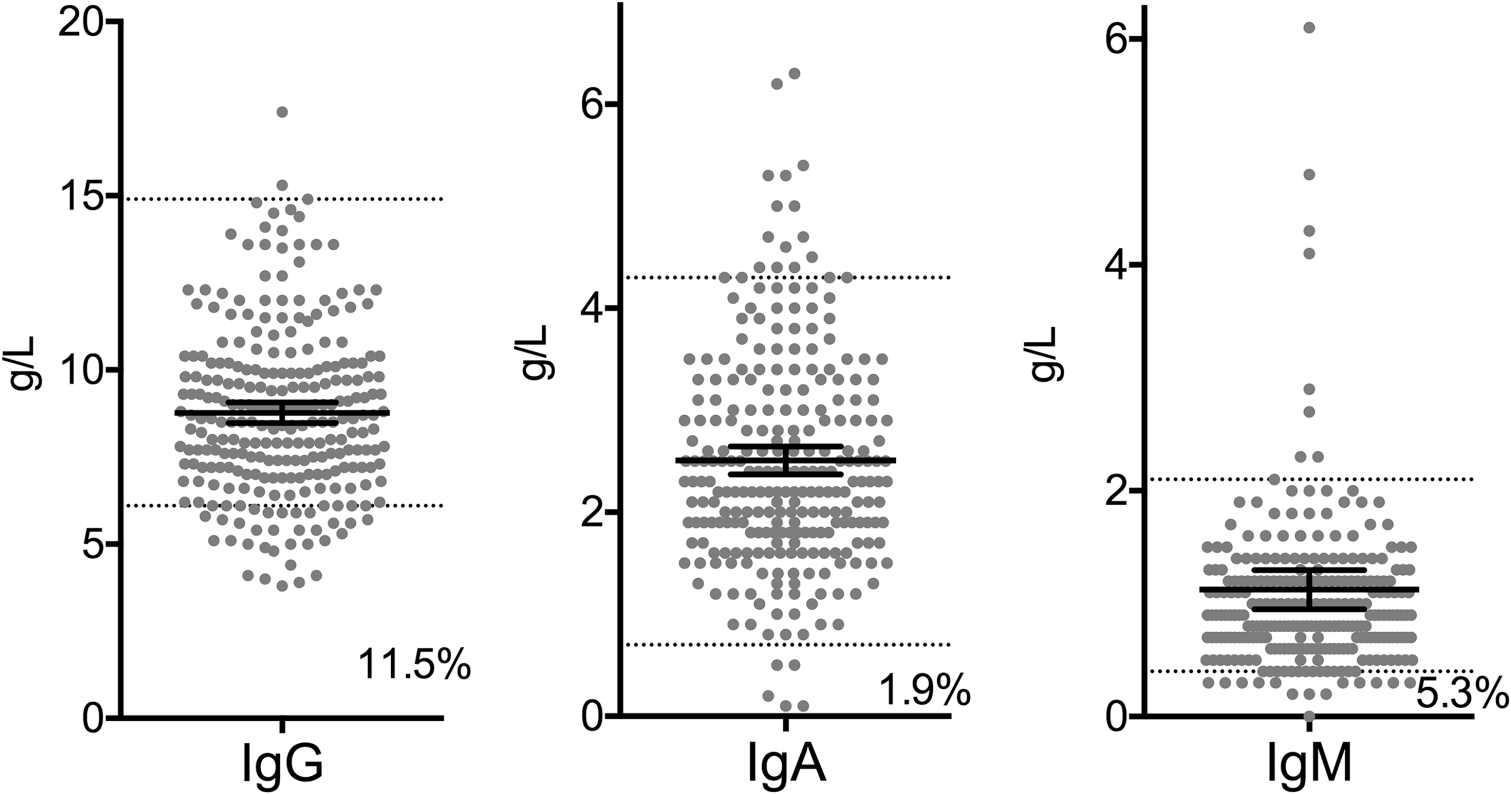
Plasma concentration of immunoglobulins in 262 patients with COPD stage II–IV. Black lines indicate mean and 95% CI, dotted line indicates upper and lower reference values. Numbers in graph indicate percentage of patients with values below reference.

Thirty patients (11.5%) had IgG levels below reference values. These patients are hereafter termed the hypogamma-COPD group. Moreover, 14 patients (5.3%) had an IgM value below reference values, while five patients (1.9%) had low IgA (Figure 1). Only one patient had low IgG and low IgA, while four patients had low IgG and low IgM.

There was no significant difference between hypogamma-COPD patients and the others regarding gender, smoking history or comorbidities such as heart disease, rheumatic disease, osteoporosis, diabetes or kidney disease. Notably, the patients with hypogamma-COPD had lower BMI (21.1 vs 24.4, p<0.001) and a higher St. George total score (67.4 vs 54.7, p<0.001, Table 1). There were no significant differences in inflammatory parameters, such as CRP (mean 5.2 mg/mL vs 7.5 mg/mL, NS), leukocytes (mean 9.0 x 10 ^9^/L vs 8.3 x 10 ^9^/L, NS), and eosinophils (mean 0.2 vs 0.2, NS) between hypogamma-COPD and non-hypogamma-COPD. There was no difference in blood albumin (not shown).

**Table 1:** Patient Descriptives

	Hypogamma-COPD (n=30)	Non-Hypogamma-COPD (n=232)	P-value
Female n (%)	19 (63.3)	118 (50.9)	P=0.198
Age	60.2 (7.73)	63.5 (8.89)	P=0.058
BMI	21.1 (3.06)	24.4 (4.66)	P<0.001
Currently smoking n (%)	3 (10.0)	58 (25.0)	P=0.067
Tobacco use (years)	38.0 (8.16)	40.0 (11.54)	P=0.249
Alpha 1-antitrypsin deficiency n (%)	4 (14.8)	21 (9.9)	P=0.432
Heart disease n (%)	5 (16.7)	59 (25.4)	P=0.293
Rheumatic disease n (%)	0	19 (8.2%)	P=0.104
Osteoporosis n (%)	3 (10.0)	20 (8.6)	P=0.802
Kidney disease n (%)	1 (3.3)	6 (2.6)	P=0.811
Diabetes n (%)	2 (6.7)	16 (6.9)	P=0.963
Bowel disease n (%)	3 (10.0)	7 (3.0)	P=0.060
Cancer n (%)	1 (3.3)	9 (3.9)	P=0.883

Among patients with hypogamma-COPD, 76.7% had GOLD stage IV while among patients with non-hypogamma-COPD only 43.1% had GOLD stage IV (p=0.001). Of all patients with COPD grade IV, 18.7% had hypogamma-COPD. Correspondingly, only 6.7% of the patients with hypogamma-COPD had GOLD stage II while 33.6% of the non-hypogamma-COPD patients had GOLD stage II (p=0.002). Applying the 2011 GOLD criteria, we found that 86.2% of the hypogamma-COPD patients were in group D (high risk – more symptoms) while among the non-hypogamma-COPD 57.5% were in group D (p=0.003). There was no significant difference between those with or without hypogamma-COPD regarding the distribution between groups A, B, and C (not shown).

A higher proportion of patients with hypogamma-COPD had dyspnea equivalent to an MMRC grade 4 (62.1% vs 30.1%, p=0.001, Table 2), and they also had shorter walking distance in the 6MWT (310.7 vs 387.6 meters, p=0.007). There were no differences between the patients with hypogamma-COPD and the others in the occurrence of chronic bronchitis (daily coughing). There was a trend toward higher occurrence of hyperinflation on chest X-ray among hypogamma-COPD patients (89.3% vs 73.3%, p=0.063, Table 3).

**Table 2:** MMRC Scale

	Hypogamma-COPD n=30	Non-Hypogamma-COPD n=232	P-value
Grade 0 n (%)	1 (3.4)	18 (8.0)	P=0.383
Grade 1 n (%)	3 (10.3)	54 (23.9)	P=0.099
Grade 2 n (%)	1 (3.4)	45 (19.9)	P=0.030
Grade 3 n (%)	6 (20.7)	41 (18.1)	P=0.739
Grade 4 n (%)	18 (62.1)	68 (30.1)	P=0.001

**Table 3:** Clinical Presentation and Treatment of COPD

	Hypogamma-COPD (n=30)	Non-Hypogamma-COPD (n=232)	P-value
Years since COPD diagnosis	10.3 (6.69)	7.4 (6.11)	P=0.019
Cough daily for last three months n (%)	9 (31.0)	83 (38.1)	P=0.461
Hyperinflation on X-ray n (%)	25 (89.3)	140 (73.3)	P=0.067
Numbers of COPD admissions last year median (IQR)	1.5 (0.75–3.25)	0.0 (0.0–1.0)	P<0.0001(MW)
Number of prednisolone treatments last year median (IQR)	3.0 (1.25–5.0)	0.0 (0.0–2.0)	P<0.0001(MW)
Number of antibiotic treatments last year median (IQR)	2.0 (1.0–5.0)	1.0 (0.0–2.0)	P=0.0001(MW)
Use of steroids n (%)^a^	30 (100)	170 (76.2)	P=0.003
Use of inhaled beta 2 agonist n (%)	27 (90.0)	152 (68.2)	P=0.014
Use of inhaled anticholinergics n (%)	30 (100)	186 (81.9)	P=0.011
Use of leukotriene antagonist n (%)	7 (25.0)	12 (5.3)	P<0.001
Use of theophylline n (%)	14 (46.7)	26 (11.4)	P<0.001
Prednisolone treatment n (%)	13 (46.4)	31 (13.7)	P<0.001
Oxygen therapy n (%)	18 (60.0)	63 (27.2)	P<0.001
FEV1% predicted	26.7 (14.46)	40.0 (19.29)	P<0.001
FVC (L)	1.9 (0.71)	2.4 (0.89)	P=0.002
DLCO (mmol/(min × kPa))	3.5 (1.98)	4.4 (3.50)	P=0.198
RV (L)	4.5 (2.05)	5.0 (6.09)	P=0.794
TLC (L)	7.5 (1.73)	7.0 (1.89)	P=0.416
6 minute walk test (m)	311 (123.7)	388 (130.5)	P=0.007
SGRQ score total	67.4 (15.01)	54.7 (17.99)	P<0.001

**Notes:** ^a^Either steroid in combined inhaler or alone. All categorical data shown as number and percentage, P-value calculated using Chi-squared test. All continuous data stated as mean (SD) and P-values calculated using Student's *t*-test unless otherwise stated. **Abbreviations:** BMI, body mass index; MW, Mann–Whitney test; SD, standard deviation; IQR, interquartile range; FEV1, forced expiratory flow in one second; FVC, forced vital capacity; DLCO, diffusion capacity for carbon monoxide; RV, residual volume; TLC, total lung capacity.

**Figure 2:**
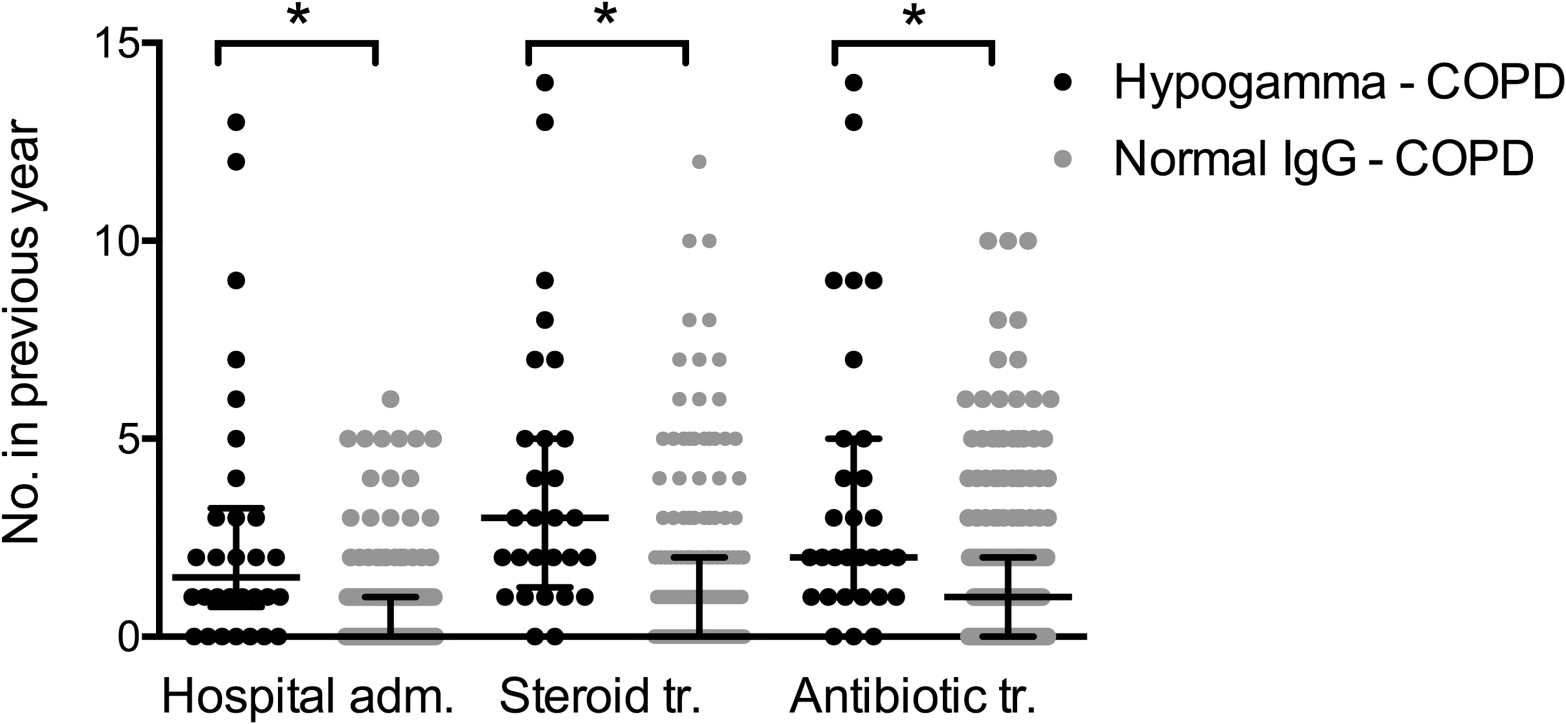
Acute exacerbations of COPD in hypogamma-COPD vs normal-IgG-COPD. Black dots: COPD patients with IgG <6.1 g/L, grey dots: COPD patients with normal IgG levels. Black lines indicate median and interquartile range. *Indicates significant difference (p<0.0001, Mann–Whitney test). Hospital adm., hospital admissions; Steroid tr., oral steroid treatments; Antibiotic tr., antibiotic treatments.

**Figure 3:**
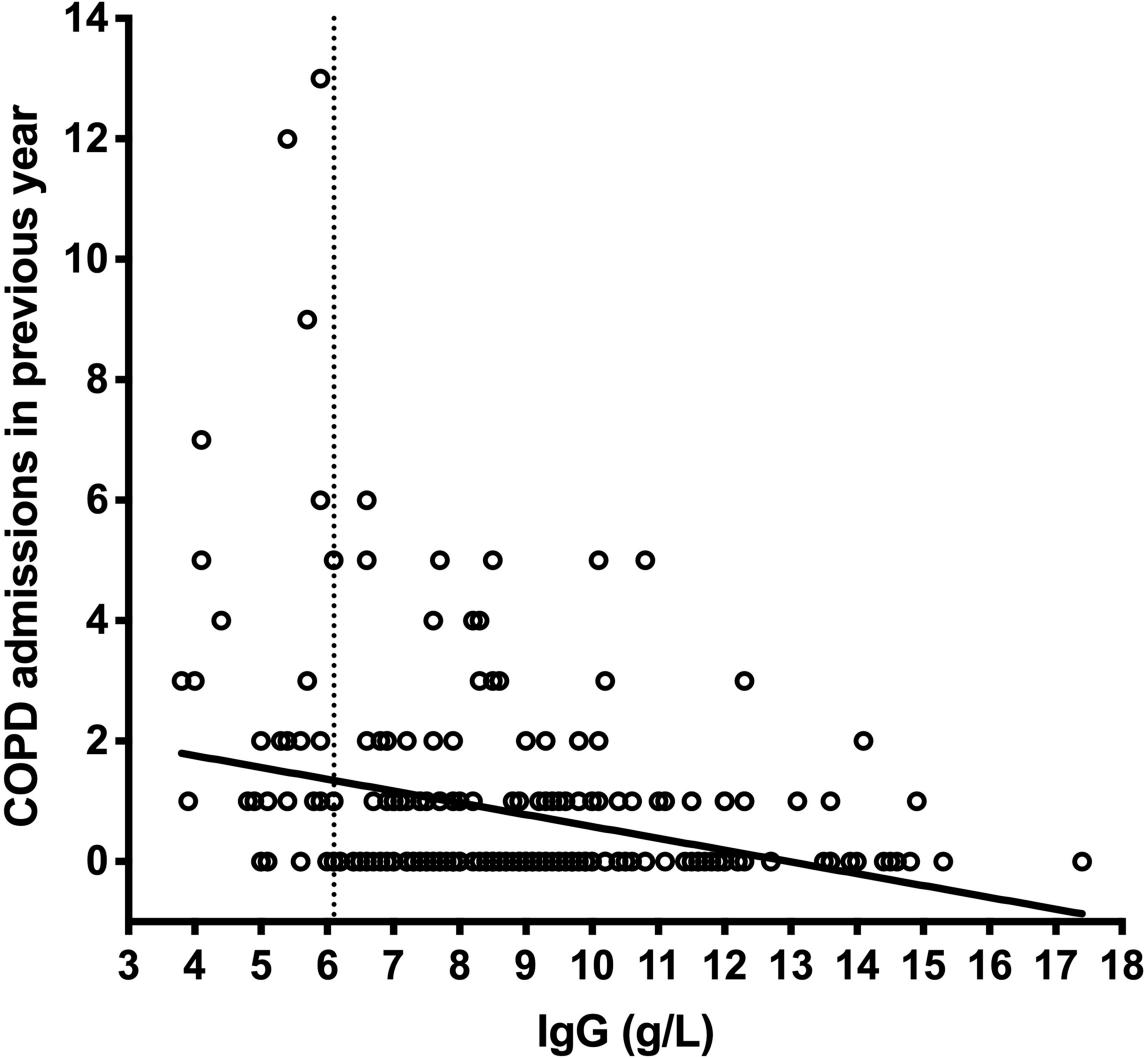
COPD admissions in previous year by serum IgG levels. There was a significant non-parametric correlation between number of COPD admissions in the previous year (the year preceding inclusion) and the serum levels of IgG measured upon inclusion (Spearman's test p<0.0001).

Importantly, patients with hypogamma-COPD had significantly more COPD-related hospital admissions (2.8 vs 0.6, p=0.002), number of prednisolone tapers (3.9 vs 1.2, p<0.001), and antibiotic treatments (3.7 vs 1.5, p=0.003) in the year preceding inclusion (Figure 2). Patients with hypogamma-COPD also had lower FEV1% predicted (26.7 vs 40.0, p<0.001). A higher proportion used inhaled steroids (100% vs 76.2%, p=0.003), inhaled beta2-agonists (90% vs 68.2%, p=0.014), and oral prednisolone at inclusion (46.4% vs 13.7%, p<0.001, Table 3). There was a significant non-parametric correlation between serum levels of IgG and number of hospital admissions (Figure 3).

When analyzing only patients with GOLD stage IV, there was still a higher number of COPD admissions (3.4 vs 0.9, p=0.004), prednisolone treatments (4.5 vs 1.8, p=0.004), and antibiotic treatments (4.3 vs 2.1, p=0.015) in the preceding year in the patients with hypogamma-COPD compared to those with non-hypogamma-COPD. The use of oral prednisolone at inclusion was also higher (52.4% vs 20.4%, p=0.002), while differences in use of inhaled steroids did not reach statistical significance (100% vs 86.7%, p=0.064).

All included patients were invited to a follow-up after one year. We found that there was good agreement between reported number of exacerbations in the year preceding inclusion and in the prospective registration in the following year (R=0.68, p<0.0001). Importantly, the hypogamma-COPD patients again reported to have had significantly more hospital admissions (1.7 vs 0.4, p=0.026), prednisolone treatments (2.5 vs 1.1, p=0.006), and antibiotic treatments (2.7 vs 1.3, p=0.029) in the year following inclusion compared to the non-hypogamma COPD patients, confirming the observations made at inclusion.

Pneumococcal antibody levels were measured in 204 patients. Patients with hypogamma-COPD had significantly lower pneumococcal antibody levels (11.04 U/mL vs 15.28 U/mL, p=0.04). Of note, only 44.2% of the included patients reported to have received pneumococcal vaccine despite current vaccine recommendations. There was a higher trend toward positive vaccine status among the patients with hypogamma-COPD (73.9% vs 52.9%, p=0.057).

In the 180 patients where IgG subclasses were measured, we found that 29 (16.1%) had low IgG1, while 40 (22.2%) had low IgG2. Similar to the pattern seen when considering total IgG levels, we found that compared to patients with high or normal IgG2, the patients with low IgG2 had a significantly higher number of COPD admissions in the preceding year (1.9 vs 0.7, p<0.001), as well as number of antibiotic treatments (2.9 vs 1.7, p=0.01) and prednisolone treatments (3.0 vs 1.5, p=0.002).

An earlier study of this patient cohort found three latent classes regarding physical and psychological symptoms. Of those with hypogamma-COPD, 3.3% were in the class reporting a low score on all symptoms, while 46.7% of the hypogamma-COPD patients reported a low score on psychological symptoms but high on physical symptoms, and finally 50.0% reported a high score on psychological and physical symptoms. There was no significant difference in the distribution of latent class groups when comparing hypogamma-COPD patients to those with non-hypogamma COPD.

In the multivariate regression analysis, we found that hypogamma-COPD was an independent predictor for number of prednisolone treatments (OR = 4.3, p=0.003) and COPD admissions (OR = 5.1, p=0.001).

**Figure 4:**
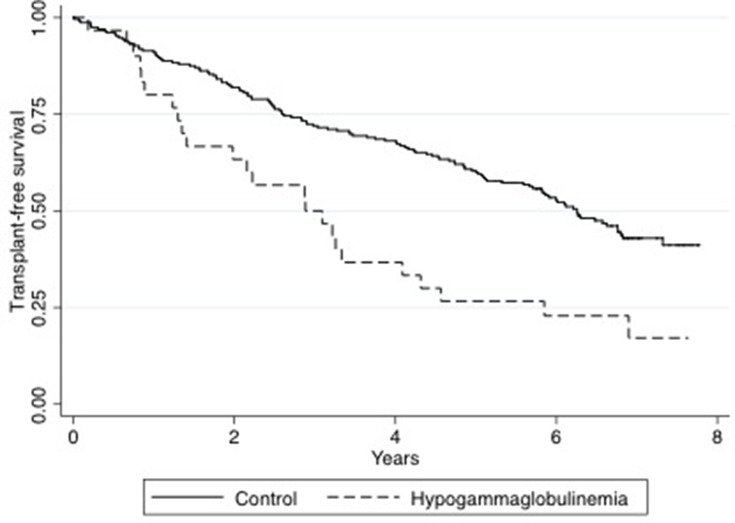
Survival after inclusion in hypogamma-COPD vs normal-IgG-COPD. The Kaplan–Meyer plot shows transplant-free survival of all included patients from time of inclusion to five years after inclusion of the last patient. Black line: normal-IgG-COPD. Dotted line: hypogamma-COPD. Survival compared using Log-rank test (p=0.0003).

Survival status (transplant-free survival) for all included patients was assessed five years after inclusion of the last patient, ie, in 2017. Of all patients included, 27.3% were dead five years after inclusion. Notably, we found that those with hypogamma-COPD had significantly poorer transplant-free survival than the controls using Log Rank analysis (p=0.0003) (Figure 4).

## Discussion

In this study of patients with stable COPD, 30 patients (11.5%) had IgG below reference values. Compared to the other COPD patients, subjects with hypogamma-COPD had lower FEV1% predicted and thus a higher proportion had GOLD stage IV. They had significantly more COPD admissions, antibiotic treatments, and prednisolone treatments in the preceding year. Also, a larger fraction of the hypogamma-COPD patients used medication such as inhaled steroids and oral prednisolone at inclusion compared to the other COPD patients, and, importantly, they had lower survival.

The prevalence of low IgG in COPD patients has not previously been studied in a large cohort. In the patient cohorts analyzed by Leitao Filho et al, 18–20% had reduced levels of one or more subclasses.^13^ In a study of 40 patients waiting for lung transplantation, pre-transplant values showed that six of 13 patients with COPD had mild hypogammaglobulinemia.^22^ In another study of 15 patients with corticosteroid-dependent COPD, five COPD patients had low IgG.^23^ Why such a high percentage of the COPD population have hypogammaglobulinemia is not clear. As discussed by others, it could be secondary, either as a side effect of medications or through some unknown disease mechanism in COPD.^24^,^25^ Alternatively, it could be a manifestation of an immune dysfunction not secondary to COPD, leading to more frequent exacerbations, possibly related to infections, and consequently a more rapid disease progression.

The data obtained in this study do not allow conclusions about whether hypogammaglobulinemia is the cause or the consequence of having an aggressive, exacerbating COPD phenotype. It is possible that the hypogammaglobulinemia observed is related to a high degree of systemic inflammation in the more severely ill patients, independently of treatment given, and it is known that frequent exacerbations may be caused by chronic inflammation.^26^ However, in our study we found no difference in CRP or white blood cell counts at inclusion when comparing patients with hypogamma-COPD and those with normal IgG levels.

In this study, ¾ of the hypogamma-COPD patients had GOLD stage IV. Earlier studies have shown a relation between frequent exacerbations and faster decline in FEV1, as well as mortality.^27^,^28^ Although we found that the association between hypogammaglobulinemia and having frequent exacerbations was independent of the current FEV1% predicted value, it is conceivable that previous exacerbations may have caused the increased bronchial obstruction in the hypogamma-COPD patients. Conversely, it is also possible that having a rapidly progressive type of COPD with frequent exacerbations can lead to an increased use of medication, such as steroids in various forms, which may enhance the tendency toward hypogammaglobulinemia.^29^ The relationship between inhaled corticosteroids, either alone or combined, and exacerbations is unclear.^30^,^31^ In a study of 100 patients with asthma and either inhaled corticosteroid alone, or in combination with an oral corticosteroid, no patients with inhaled corticosteroids alone had hypogammaglobulinemia. In contrast, patients with oral corticosteroids >12.5 mg/day for at least one year had reduced levels of serum IgG.^32^ Moreover, in a study of COPD patients with or without steroid therapy, it was observed that significantly lower levels of IgG were found in the patients on steroid therapy, although oral or inhaled steroids were not distinguished.^33^ In the present study, all of the hypogamma-COPD patients used ICS and significantly more patients with hypogamma-COPD used oral prednisolone at inclusion. This suggests that steroid use may be associated with hypogammaglobulinemia in COPD, but the causal relationship is still unclear.

Another known factor related to hypogammaglobulinemia is smoking. Lower plasma levels of IgG have been found in smokers compared to non-smokers but no correlation between low IgG and recurrent exacerbations could be demonstrated.^34^–^36^ In the present study we found no relation between smoking status and hypogamma-COPD.

The observation that the patients with hypogamma-COPD have a higher rate of infections may indicate that preventive measures are warranted. However, we found that although the patients with hypogamma-COPD tended to report a higher pneumococcal vaccination rate, their titers of pneumococcal antibodies were lower. Whether this is because of a reduced immunological response to vaccines or some other mechanisms is unclear, but this observation may be of importance when designing preventive measures in COPD, and should be subjected to further studies.

When following the included patients for at least five years, we found that the hypogamma-COPD patients had significantly lower transplant-free survival. Whatever the causal relationship between severe COPD and hypogammaglobulinemia may be, this observation may suggest that immunoglobulin replacement therapy may be a feasible target for interventional studies in patients with COPD who have low levels of IgG, particularly considering the poor prognosis and the scarcity of other effective treatment options in this common disease.^24^,^25^

The study has some limitations. First, of the total study population, for five individuals (1.8%) blood tests were missing, including IgG. These patients came from three of the five study hospitals, and had GOLD stage II or III. Second, it might be argued that some unreliability is associated with the self-reporting of data by the patients, as data regarding COPD admissions, antibiotics, and prednisolone cures were patient-reported through questionnaires. However, it has previously been demonstrated that the agreement between self-reported medical information and data assessed from medical charts is good in patients with diabetes and other well-known chronic diseases.^37^,^38^ Moreover, in the present study there was good agreement between reported number of exacerbations in the year preceding inclusion and number of exacerbations registered prospectively in the year following inclusion. Unfortunately, the precise time of exacerbation was not registered, and the temporal relationship between IgG measurements and exacerbation could therefore not be calculated. Also, the analyses were limited by the absence of information about cause of death. Finally, some of the included patients received a lung transplant. In the survival analyses, this was treated as a competing risk, but we do not know how these patients would have affected the results had they not received a transplant. There is, however, a consensus not to perform transplant unless a survival of less than two years may be assumed.^39^

In conclusion, we found that 11.5% of the COPD patients included in this study had reduced serum levels of IgG, and that the occurrence of hypogammaglobulinemia in those with COPD GOLD stage IV approached 20%. The occurrence of hypogammaglobulinemia was related to use of inhaled and oral steroids, but independently of these factors, we found that hypogammaglobulinemia predicted an increased number of exacerbations and hospitalizations and that hypogammaglobulinemia is associated with impaired long-term survival. Our observations suggest that both the function of specific elements of the immune system, such as B-cells, and the clinical consequences and effects of hypogammaglobulinemia on survival in patients with COPD should be studied. Furthermore, our observations suggest that the possibility of interventional studies using intravenous immunoglobulins to prevent exacerbations in selected COPD patients should be considered.
